# Unraveling the impact of upfront chemotherapy and proton beam therapy on treatment outcome and follow-up in central nervous system germ cell tumors: a single center experience

**DOI:** 10.3389/fonc.2023.1259403

**Published:** 2023-10-04

**Authors:** Giada Del Baldo, Sabina Vennarini, Maristella Toniutti, Rachid Abbas, Stefano Lorentini, Eleonora Piccirilli, Antonella Cacchione, Giacomina Megaro, Valentina Di Ruscio, Maria Antonietta De Ioris, Andrea De Salvo, Giulia Albino, Sabrina Rossi, Giovanna Stefania Colafati, Andrea Carai, Angela Mastronuzzi

**Affiliations:** ^1^ Department of Pediatric Haematology and Oncology, and Cell and Gene Therapy Bambino Gesù Children’s Hospital, IRCCS, Rome, Italy; ^2^ Department of Experimental Medicine, Sapienza University of Rome, Rome, Italy; ^3^ Pediatric Radiotherapy Unit, Fondazione IRCCS Istituto Nazionale dei Tumori, Milan, Italy; ^4^ Department of Medicine DAME-Division of Pediatrics, University of Udine, Udine, Italy; ^5^ CESP, INSERM, Université Paris Sud, Villejuif, France; ^6^ Medical Physics Department, Azienda Provinciale per i Servizi Sanitari (APSS), Trento, Italy; ^7^ Department of Diagnostic Imaging Oncological Neuroradiology Unit, Bambino Gesù Children’s Hospital, IRCCS, Rome, Italy; ^8^ Department of Neuroscience, Imaging and Clinical Sciences, University of Chieti, Chieti, Italy; ^9^ Pathology Unit, Bambino Gesù Children’s Hospital, IRCCS, Rome, Italy; ^10^ Neurosurgery Unit, Department of Neurosciences, Bambino Gesù Children’s Hospital, IRCCS, Rome, Italy

**Keywords:** CNS germ cell tumors, chemotherapy, protontherapy, outcome, side effects, pediatric brain tumors

## Abstract

**Background:**

Germ cell tumors (GCT) account for a minority of central nervous system (CNS) malignancies, highly prevalent in adolescents and young adults. Despite their aggressive biological behavior, prognosis is excellent in most cases with risk stratified treatment, consisting in a combination of chemotherapy and radiotherapy. Whole ventricular irradiation (WVI) and craniospinal irradiation, the treatment of choice for localized and metastatic disease, pose significant risk of collateral effects, therefore proton beam radiation (PBT) has been recently proposed for its steep dose fallout.

**Materials and methods:**

We report our experience in a consecutive series of 17 patients treated for CNS GCT at our Institution from 2015 to 2021.

**Results:**

Most frequent lesion location were sellar/suprasellar (35%) and bifocal germinoma (35%), followed by pineal (18%) and thalamic (12%). Two patients (12%), had evidence of disseminated disease at the time of diagnosis. At the latest follow-up all but one patient showed complete response to treatment. The only relapse was successfully rescued by additional chemotherapy and PBT. PBT was well tolerated in all cases. No visual, neurological or endocrinological worsening was documented during and after treatment. Neuropsychological evaluation demonstrated preservation of cognitive performance after PBT treatment.

**Conclusions:**

Our data, albeit preliminary, strongly support the favourable therapeutic profile of PBT for the treatment of CNS germ cell tumors.

## 1 Introduction

Central nervous system (CNS) germ cell tumors (GCT) are more prevalent among adolescents and constitute approximately 3-5% of pediatric brain tumors (1–3). These tumors typically manifest in the pineal and sellar/suprasellar regions, and although rare have the potential to metastasize (2). Histopathologically, GCTs are categorized into germinomas, which are the most common type, and non-germinomatous tumors. Considering their high sensitivity to treatment, therapeutic strategies primarily involve chemotherapy and radiotherapy. In the past, radiation treatment encompassed the entire brain and spinal cord through craniospinal irradiation (CSI), but this approach was abandoned due to well-known short- and long-term side effects (4). Presently, radiation therapy is limited to the whole ventricular system (WVI), with a targeted boost on the primary site in localized germinoma cases, while CSI is reserved for metastatic disease (5). Neoadjuvant chemotherapy plays a crucial role in reducing tumor size, subsequently minimizing the radiation field required (6, 7). The current prognosis for germinoma, including metastatic disease, exceeds 90% in terms of overall survival rates (8, 9). Given the high rates of successful treatment, the primary objective now focuses on improving the quality of life by minimizing long-term complications. Well-documented side effects of CNS radiation in the pediatric population include cognitive impairments, neuroendocrine dysfunctions, sensorineural hearing loss, and second malignancies (10–13). Furthermore, craniospinal irradiation poses additional risks of cardiac and pulmonary dysfunction, hypothyroidism, gastrointestinal symptoms, growth impairment, and infertility. Consequently, recent efforts have aimed to limit side effects, minimize the target volume, and reduce radiation doses to surrounding normal tissues. Various radiation modalities are available for treating CNS GCTs, with proton beam therapy (PBT) emerging as a promising option. However, the clinical experience with PBT in this context remains limited. Therefore, the objective of our study is to retrospectively analyze our institution’s cohort of pediatric patients with CNS GCTs who received chemotherapy followed by PBT, providing additional insights into survival rates and long-term complications.

## 2 Materials and methods

### 2.1 Patient population

We conducted a retrospective analysis of consecutive patients diagnosed with CNS germinoma at Bambino Gesù Children’s Hospital in Rome between May 2015 and April 2021. The choice of 2015 as the study’s starting point was based on the availability of PBT for clinical use in Italy.

The inclusion criteria for this study were as follows: patients between 0 and 25 years of age, histological confirmation of germinoma or bifocal localization without necessity of biopsy, and treatment with PBT. The medical records of the patients were reviewed, including demographic information, details of any surgical procedures performed, treatment for hydrocephalus (if applicable), specifics of chemo and radiation therapy, visual and endocrinological assessments at the different timepoints (diagnosis, after chemotherapy and PBT, every 6-month during follow-up). Neuropsychological assessments were conducted at diagnosis, before and after PBT, and 12 months after PBT. Additionally, data on patient outcomes at the last follow-up were collected.

This study was approved by the Institutional Review Board (IRB) of Bambino Gesù Children’s Hospital and conducted in accordance with the principles outlined in the Helsinki Declaration (RAP-2023-0008). Written informed consent was obtained from all patients or their legal guardians.

### 2.2 Biochemical assessment

At the time of diagnosis, all patients underwent measurement of alpha-fetoprotein (AFP) and human chorionic gonadotropin (hCG) levels in both serum and cerebrospinal fluid (CSF).

For the determination of AFP and hCG levels in CSF samples and serum, the ADVIA Centaur^®^ XPT Immunoassay System (Siemens Healthineers Diagnostics, Erlangen, Germany) was utilized.

The AFP and hCG assays employed a two-site sandwich immunoassay method, utilizing direct chemiluminometric technology. This approach involved the use of two antibodies in fixed quantities (14).

### 2.3 Radiological assessment: diagnosis and treatment response

All patients included in the study underwent a standardized MRI protocol at our institution using a 3T scanner (Siemens Magnetom Skyra, Erlangen, Germany) to comprehensively evaluate the entire neuroaxis. The protocol encompassed several sequences, including axial and coronal T2-weighted turbo spin echo (TSE), fluid-attenuated inversion recovery (FLAIR), diffusion-weighted imaging (DWI), susceptibility-weighted imaging (SWI), pre- and post-contrast axial and 3D T1-weighted sequences of the brain, and T1- and T2-weighted TSE sequences of the spine and spinal cord on sagittal and axial planes. Additionally, high-resolution T1- and T2-weighted sequences were acquired specifically to assess midline structures, such as the pineal and sellar/suprasellar regions. In cases where children were uncooperative, general anesthesia was administered during the MRI scans.

Radiological images were evaluated at four different time points: at diagnosis, after chemotherapy, after PBT, and every three months during the follow-up period. High-resolution images were used at each time point to measure the volume of the pineal gland and/or the maximum thickness of the pituitary stalk (in the sagittal plane) when applicable.

### 2.4 Surgery

The indication for surgery was determined through discussions in a multidisciplinary tumor board for all cases. Surgical biopsy was recommended for sellar/suprasellar or pineal single lesions with low detection of hCG and AFP in serum and/or CSF, and avoided in bifocal cases. All surgical procedures were carried out by a specialized pediatric neurosurgical team. The standard biopsy method involved a robot-assisted (Rosa, Medtech) endoscopic (Decq, Storz) transventricular technique using a precoronal approach.

For pituitary stalk lesions that did not extend to the floor of the third ventricle, a pterional craniotomy and microsurgical technique were employed. Bipolar coagulation of diencephalic structures was avoided, and a longitudinal incision was made in the enlarged stalk to obtain pathological tissue while minimizing disruption of nervous fibers and the hypophyseal portal system. In cases of pure intrasellar lesions confined below the plane of the diaphragma sellae, the preferred approach was the transnasal trans-sphenoidal endoscopic method.

In instances where hydrocephalus was present due to aqueductal obstruction by a pineal region mass, an endoscopic third ventriculostomy was performed immediately before the biopsy procedure using a single trajectory.

### 2.5 Histopathological characterization

Histological examination based on the morphological and immunophenotypic characteristics of the tumor biopsy was conducted for all cases. Immunohistochemistry (IHC) was performed on formalin-fixed paraffin-embedded sections using an automated immunostainer (Dako Omnis). The IHC panel included the assessment of SALL4, OCT3/4, PLAP, and CD117 expression. To ensure accuracy and consistency, all tumor tissue samples were centrally reviewed.

### 2.6 Ophthalmologic assessment

Visual performance data were gathered during several time points: at the time of diagnosis, after chemotherapy, after PBT, and every six months during follow-up. The assessments conducted encompassed visual acuity (VA), optical coherence tomography (OCT), and visual field (VF) testing. Snellen charts were employed to evaluate VA (15). The measurement of retinal nerve fiber layer thickness (RNFL) using OCT was utilized to predict visual loss. A reduction of greater than 10% in one or more quadrants or the overall average from the baseline was considered indicative of visual loss (16). VF testing was conducted using age-appropriate methods and administered by a pediatric ophthalmologist to cooperative patients. The methods employed included a behavioral VF screening test, Humphrey visual field analyzer, semi-automatic static Peritest, or Goldmann kinetic perimetry (17–19).

### 2.7 Endocrinological assessment

Endocrinological data were obtained at different time points: upon diagnosis, post-chemotherapy, after PBT, and every six months during the follow-up period. Clinical and auxological data were reported. The evaluation of pituitary function involved assessing deficiencies in gonadotropins, thyrotropin, corticotropin, growth hormone (GHD), and diabetes insipidus. Retrospectively, biochemical and hormonal data were collected, including IGF-I, IGFBP-3, FT4, TSH, FSH, LH, testosterone, 17-beta-estradiol, ACTH, cortisol, glucose, serum and urinary electrolyte levels, as well as plasma and urine osmolarity. Hormonal detection was measured using the chemiluminescent immunometric assay (Immulite 2000 XPi, Siemens). The endocrine evaluation encompassed provocative tests to investigate hypothalamic-pituitary function, as described previously (20). Information regarding hormonal replacement treatment was documented for all patients, if applicable.

### 2.8 Cognitive evaluation

Cognitive assessment took place at various stages: prior to the commencement of treatments at the time of diagnosis, both before and after PBT, with a one-year interval between each assessment, and finally, during the follow-up phase, one year after the last assessment. The choice of appropriate scales for evaluation depended on the patient’s age and were validated for clinical use, such as the Weschler scales, including the WISC or WAIS 4th edition (21). These scales provided an estimation of overall intellectual ability, known as the full-scale IQ, along with four composite scores: verbal comprehension index (verbal skills), perceptual reasoning index (nonverbal reasoning), working memory index (working memory, short-term memory, sustained attention, and auditory processing), and processing speed index (visual-motor coordination, attention, concentration, and speed of mental processing). For this study, only the scores representing the overall cognitive level were reported (22).

### 2.9 Chemotherapy

The chemotherapy regimen used in this study was selected based on the indication of The International Society of Pediatric Oncology (SIOP) CNS GCT II protocol. It consisted of four courses of chemotherapy, with two cycles of carboplatin (900 mg/m^2^ on day 1) and etoposide (100 mg/m^2^ on day 1-3), alternating with two cycles of ifosfamide (1800 mg/m^2^ on day 1-5) and etoposide (100 mg/m^2^ on day 1-3), administered every 21 days. All medications were administered intravenously. Toxicity assessments were conducted through clinical evaluations, laboratory values, and imaging and were recorded following CTCAE 5.0 criteria (23).

### 2.10 Proton beam therapy

Prior to undergoing irradiation, all patients participated in multidisciplinary discussions. To define tumor volumes and determine the prescribed doses for localized (mono-bifocal) and disseminated forms of GCT, guidelines outlined in of the European protocol SIOP CNS GCT II were followed. This process involved conducting a CT examination and creating a personalized immobilization system. Subsequently, specific MRI sequences were acquired, including T2, T1, and T1 weighted images with the use of contrast medium, in various spatial planes. These images were utilized to prepare the treatment volumes. The Gross Tumor Volume of the tumor bed (TB-GTV) encompassed both the initial disease volume and any residual disease remaining after chemotherapy. An additional margin of 0.5 cm was added to obtain TB-CTV, and an additional 0.3 cm margin was applied to derive TB-PTV. The TB-PTV volume received sequential boost irradiation following the completion of WVI-PTV rather than using a Simultaneous Integrated Boost (SIB) approach. In the case of disseminated forms or instances with CSF positivity, the treatment approach involved CSI, followed by boosts targeted at the primary disease site.

Proton plans were developed using a Single Field Optimization (SFO) technique. The field arrangement included three non-coplanar beam directions: a posterior field along with two oblique fields (one from the right and one from the left). This arrangement was designed to minimize the dose delivered to the hippocampus regions and temporal lobes. Special attention was given to avoid distal penumbra overlaps of the different fields in critical structures such as optic pathways and the brainstem. CSI was delivered using a rotational gantry system (24).

The proton dose was prescribed in Gy (RBE) (Gray relative biological effectiveness), with a constant relative biological effectiveness value set to 1.1. All plans were delivered using the pencil beam scanning technique (25).

During proton treatment, patients underwent clinical and hematological evaluations on a weekly basis.

The acute and late effects of PBT were assessed in accordance with the National Cancer Institute Common Toxicity Criteria (Version 5.0) (23).

### 2.11 Statistical analysis

Data entry and cleaning processes were conducted using Microsoft Excel. Data analyses were performed utilizing SAS 9.4 version software.

Evaluation of lesion volume and stalk diameter took place at diagnosis, during treatments, and during follow-up. Volumetric reduction after chemotherapy, PBT, and at the final follow-up was compared. The Friedman test was employed for analyzing the results.

Regarding endocrinological outcomes, patients were categorized into two groups: no hormonal deficits and hormonal deficiency. For ophthalmological outcomes, visual defects were divided into two groups: normal vision and impaired vision. Comparisons of endocrinological and visual outcomes were made at diagnosis, during treatments and follow up. Fisher’s exact test, two-sided tailored, was used for the comparison tests, with the alpha risk set at 5%.

In terms of cognitive assessment, IQ was evaluated at four time points: at diagnosis, before PBT, after PBT, and at the last follow-up. Results were compared using the Friedman test.

Overall survival (OS) was expressed as the median duration from the time of diagnosis to the last follow-up.

## 3 Results

### 3.1 Population characteristics

A retrospective analysis was conducted on data obtained from consecutive patients who were diagnosed with CNS germ cell tumors at Bambino Gesù Children’s Hospital in Rome. The study period spanned from May 2015 to April 2021. Through this retrospective review, we identified a total of 17 patients with primary CNS germ cell tumors, consisting of 13 males and 4 females. The median age at the time of diagnosis was 12.19 years, with a range of 9.86 to 21.42 years. **Table 1** provides a summary of the cohort characteristics.

**Table 1:** Population characteristics.

	Sex	Age (y)	Primary Site	M	Surgery	Relapse
**1**	M	13,75	Pineal	No	Biopsy+ETV	No
**2**	M	12,15	Pineal	No	Biopsy+ETV	No
**3**	M	21,41	Bifocal	No	No	Yes
**4**	M	11,20	Sellar/suprasellar	No	Biopsy	No
**5**	M	17,84	Bifocal	No	No	No
**6**	M	18,34	Multifocal	No	Biopsy	No
**7**	M	11,39	Bifocal	Yes	DVE	No
**8**	M	9,96	Sellar/suprasellar	No	Biopsy	No
**9**	M	19,66	Left thalamus-mesencephalic area	No	Biopsy	No
**10**	F	12,19	Suprasellar	No	Biopsy	No
**11**	M	9,86	Pineal	No	Biopsy+ETV	No
**12**	M	16,96	Bifocal	No	No	No
**13**	M	14,23	Sellar/suprasellar	No	Biopsy	No
**14**	F	10,45	Bifocal	No	No	No
**15**	F	9,86	Suprasellar	No	Biopsy	No
**16**	F	10,07	Sellar	No	Biopsy	No
**17**	M	14,09	Bifocal	Yes	No	No

All 17 cases displayed normal levels of AFP and hCG, as observed in both serum and CSF samples. Regarding the primary lesion sites, six patients (35%) had sellar/suprasellar involvement, six (35%) presented with bifocal germinoma, three (18%) had pineal lesions, one (6%) exhibited a lesion in the left thalamus-mesencephalic region, and one (6%) displayed evidence of multifocal disease, affecting regions such as the suprasellar region, pineal region, frontal regions, and corpus callosum. At the time of diagnosis, metastatic disease was observed in two patients (12%), both of whom displayed ventricular dissemination.

### 3.2 Surgical details

At the initial presentation, hydrocephalus was observed in six out of 17 patients. Among them, three patients underwent Endoscopic Third Ventriculostomy (ETV) and one patient required external shunt placement to manage the condition. The remaining two cases did not necessitate a neurosurgical approach for hydrocephalus management.

Biopsy procedures were performed in 11 cases (64%), with eight cases conducted using the endoscopic technique and three cases via craniotomy. No postoperative complications were detected following the neurosurgical procedures.

### 3.3 Treatment

All 17 patients in the study received neoadjuvant chemotherapy followed by PBT. The mean duration of chemotherapy was 76.18 days (SD ± 8), and the mean duration of PBT was 34.41 days (SD ± 11).

Chemotherapy was administered also in two cases of patients with disseminated disease in view of the high disease burden and clinical conditions at onset.

Among the treated patients, 15 had localized disease and received a dose of 24 Gy (RBE) on the whole ventricular irradiation-planning target volume (WVI-PTV). Subsequently, a sequential boost of either 16 Gy (RBE) or 30.4 Gy (RBE) was administered on the tumor bed-planning target volume (TB-PTV), depending on the presence or absence of a residual teratoma component (targeting the pure germinomatous component) post-chemotherapy. The total dose administered was either 40 Gy or 54.4 Gy (RBE) with a fractional dose of 1.6 Gy (RBE)/fraction.

To provide further details, among the cases with localized disease, 6 patients received a boost specifically targeting the pineal region, 2 patients received a boost in the sellar-suprasellar region, and 1 patient received a boost in the thalamo-mesencephalic region. For the remaining cases, a boost was not administered due to complete disease remission, as confirmed by post-chemotherapy radiological re-evaluation. Two patients initially had disseminated disease and received a dose of 24 Gy (RBE) in the craniospinal axis, along with boosts up to 40 Gy (RBE) and 54.4 Gy (RBE) at the primary disease sites. The decision to administer these doses was based on the presence or absence of a teratomatous component. Additionally, a patient with bifocal germinoma experienced disease recurrence and underwent re-irradiation at a total dose of 40 Gy (RBE), with 24 Gy (RBE) delivered in the craniospinal axis and an additional dose of 16 Gy (RBE) specifically targeting the hypothalamic-pituitary region.

During chemotherapy, no severe infections or unexpected toxicities were reported. No acute side effects were observed during PBT, and all patients completed the treatment without interruptions.

### 3.4 Radiological response

**Figure 1:**
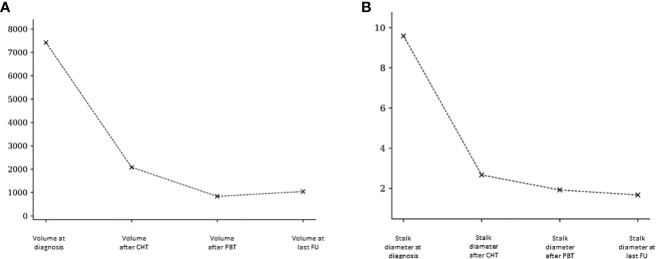
Pineal volumetric reduction **(A)** and stalk diameter reduction **(B)** after chemotherapy, after PBT and at last follow-up.

We examined the lesion volumes and stalk diameters at different stages: diagnosis, post-chemotherapy, post-PBT, and during follow-up. The trends of volume and stalk reduction are depicted in **Figure 1** and **Table 2**.

**Table 2:** Mean and median pineal volume and stalk diameter at the diagnosis, after chemotherapy, after PBT and at last follow-up.

	N	Mean (mm3/mm)	Median (mm3/mm)	p-value
**Volume at diagnosis**	11	7418.73	1350	
**Volume after CHT**	11	2079.55	280	0.228
**Volume after PBT**	11	831.36	240	0.001
**Volume at last follow-up**	11	1039.64	140	0.001
**Stalk diameter at diagnosis**	12	9.58	6.5	
**Stalk diameter after CHT**	12	2.67	2	0.036
**Stalk diameter post PBT**	12	1.92	2	0.001
**Stalk diameter at last follow-up**	12	1.67	1.5	0.001

It is worth noting that one patient developed a growing syndrome teratoma 12 months after completing PBT.

### 3.5 Ophthalmologic assessment

Among the diagnosed patients, eight individuals (47.06%) exhibited evidence of visual impairment at the time of diagnosis, as indicated in **Table 3**. During the course of treatments, three children showed improvement in their visual condition: one after receiving chemotherapy and two after undergoing PBT. No new-onset deficits were detected. Until the latest follow-up, out of the eight patients who initially had visual defects, five experienced improvements, as illustrated in **Table 4**.

**Table 3:** Visual and hormonal impairment at diagnosis.

	NORMAL	IMPAIRED
***VISUAL ASSESSMENT***	9/17 (52.94%)	8/17 (47.06%)
***ENDOCRINOLOGICAL DEFECTS***	5/17 (29.41%)	12/17 (70.59%)

**Table 4:** Visual and hormonal impairment after treatment and at last follow-up.

	Impaired at diagnosis		Worse	Stable	Improved
**Visual assessment**	8/17 (47.06%)	after chemotherapy	0/17 (0%)	16/17 (94.12%)	1/17 (5.88%)
		after proton therapy	0/17 (0%)	15/17 (88.23%)	2/17 (11.77%)
		at last follow-up	0/17 (0%)	12/17 (70.59%)	5/17 (29.41%)
**Endocrinological Defects**	12/17 (70.59%)	after chemotherapy	0/17 (0%)	17/17 (100%)	0/17 (0%)
		after proton therapy	0/17 (0%)	17/17 (100%)	0/17 (0%)
		at last follow-up	1/17 (5.88%)	15/17 (88.23%)	1/17 (5.88%)

### 3.6 Endocrinological assessment

At the initial presentation, endocrine dysfunctions were observed in 12 patients (70.59%). Among them, 3 out of 12 were diagnosed with diabetes insipidus, and 9 out of 12 exhibited two or more defects, as indicated in **Table 3**. Throughout the course of chemotherapy and PBT, all dysfunctions remained stable. No new hormonal deficiencies were detected during the treatment phase or during follow-up surveillance.

At the last follow-up, out of the 12 patients with hypothalamic impairments at the time of diagnosis 10 demonstrated stable hormonal assessments. One patient experienced a worsening condition with the development of a new deficit, although it did not necessitate replacement therapy. Conversely, one patient showed improvement, resolving central hypogonadism and presenting only with diabetes insipidus, as shown in **Table 4**.

### 3.7 Neurocognitive outcome

A baseline neurocognitive test was conducted for all patients within this cohort. The median IQ score at the time of diagnosis was 107, with a range of 55 to 131. Among the patients, 11 out of 17 (65%) achieved scores within the normal range (80–119), 4 out of 17 (23%) obtained superior scores (≥120), and 2 out of 17 (12%) fell into the low range (≤79).

**Figure 2:**
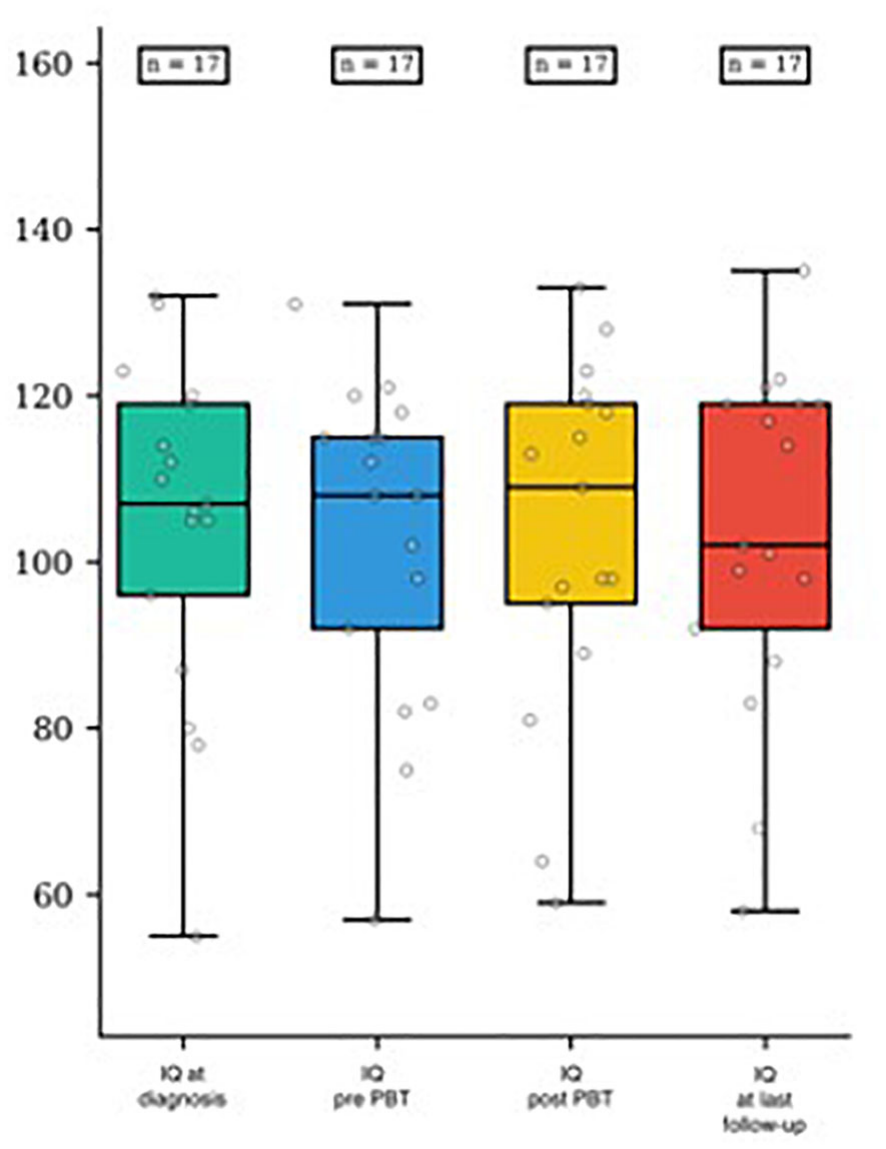
IQ score distribution at diagnosis, pre- and post-PBT and at last follow-up.

**Figure 3:**
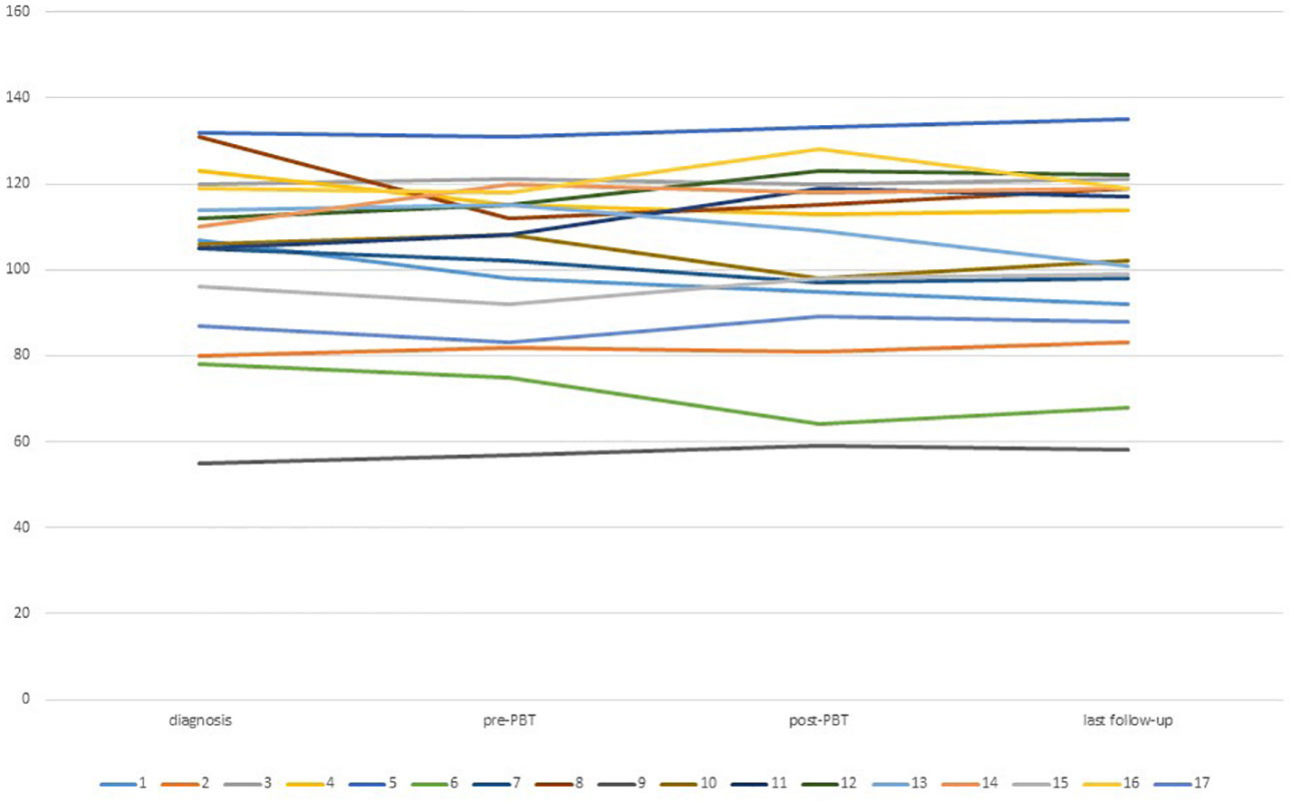
IQ trend between diagnosis and last follow up for each patient.

One patient who initially achieved high scores at diagnosis obtained normal scores after chemotherapy and maintained stability throughout the course of PBT and follow-up. Additionally, one child who initially had a normal IQ score reached a high score after PBT and at the last follow-up. However, no statistically significant changes in IQ scores were observed during treatment (p-value of 0.89). Further details of the IQ assessments can be found in **Table 5** and **Figure 2** and a plot of IQ trend for each patient in **Figure 3**.

**Table 5:** IQ trend at diagnosis, during treatments and follow up.

	MEDIAN	RANGE	Low IQ (≤79)	Normal IQ (80-119)	High IQ (≥120)
**At diagnosis**	107	55-103	2/17 (12%)	11/17 (65%)	4/17 (23%)
**Before PBT**	108	57-131	2/17 (12%)	12/17 (70%)	3/17 (18%)
**After PBT**	109	59-133	2/17 (12%)	11/17 (65%)	4/17 (23%)
**at last follow-up**	102	58-135	2/17 (12%)	11/17 (65%)	4/17 (23%)

### 3.8 Survival outcome

At the last follow-up, only one relapse occurred, accounting for 6% of the cases. The median OS for the patients included in this study was 3.5 years, with a range of 1.5 to 8.1 years. It is worth noting that one patient passed away due to an accidental cause unrelated to the disease or treatment complications.

## 4 Discussion

Germ cell tumors are rare in children, and they occur in the CNS in approximately 16% of all patients (26). Among these cases, around two-thirds are pure germinomas, which have an excellent prognosis, with a 5-year OS rate exceeding 90% (8, 9), even in cases of metastasis.

GCTs arise commonly as isolated lesions in either neurohypophyseal or pineal region. In 13-41% of cases are present at both sites and are defined as bifocal tumor (27–29). The presence of bifocal lesions in the setting AFP and HCG within normal value is considered to be pathognomonic of germinoma and the trials in North America (ACNS 1123) and Europe (SIOP CNS GCT II) included patients without necessity of biopsy. Moreover, stereotactic or endoscopic biopsy in the pineal or sellar region is not easy to perform. Biopsy is still a subject of ongoing debate in radiologically typical cases particularly given the report of non-secreting bifocal tumors that were confirmed as malignant intracranial non-germinoma germ cell tumors (30–32).

In the future other marker could help support diagnosis avoid biopsy. Placental alkaline phosphatase (PLAP) in CSF can provide a very high diagnostic value in cases of intracranial GCT, especially in pure germinomas, to the level of not requiring histological confirmation (33).

In our series, we identified a total of 17 patients with primary CNS germ cell tumors and in 35% of cases bifocal lesions were described. Biopsy procedures were performed in 11 cases (64%). Based on the considerations described above, in accordance with the European protocol SIOP CNS GCT II, we avoided biopsy in bifocal lesions, considering them as germinoma.

Given the excellent survival of this disease, the primary goal is now to improve the quality of life by reducing long-term toxicities. Germinomas are highly sensitive to chemotherapy and radiation, but chemotherapy alone is inadequate to achieve an effective and long-lasting response due to the high risk of recurrence (24–38). The current standard of care strategy involves combining both radiotherapy and chemotherapy to maximize cure rates. However, radiotherapy has been shown to have significant long-term toxicities, especially in terms of neurocognitive assessment and endocrinological impairment. Various irradiation strategies, including CSI, whole brain irradiation (WBI), WVI, and focal radiotherapy, have been attempted.

Previously, CSI followed by a boost to the tumor bed was considered the gold standard treatment, resulting in a 5-year event-free survival (EFS) rate of 97% (24). However, it was found to be overtreatment for localized cases (39). The SIOP group conducted a nonrandomized comparison study, comparing craniospinal irradiation at 24 Gy plus a focal boost of 16 Gy with the use of the SFOP CNS GCT-96 chemotherapy regimen followed by focal radiation therapy on the tumor at 40 Gy (24). There were no significant differences in OS between 125 patients treated with radiation therapy alone and 65 patients treated with combination therapy. However, a statistically significant difference in EFS favoring patients treated with radiation therapy alone was observed (97% vs. 88%, p=0.04, respectively) (24). Conversely, an excess of recurrences in the ventricles was detected. Therefore, in the SIOP CNS GCT II trial, 24 Gy WVI was introduced instead of focal radiotherapy, along with a 16 Gy boost to the tumor bed in cases of incomplete remission (CR) after chemotherapy.

Preliminary reports from the SIOP CNS GCT II trial suggest that 24 Gy WVI without a boost may be sufficient for localized germinomas with a complete imaging response post-chemotherapy (40, 41). Furthermore, a review by Rogers et al. reported comparable long-term disease control rates between localized GCTs treated with CSI and those treated with focal radiation combined with WVI (39). Considering the lower incidence of GCT spreading through the CSF compared to locoregional and ventricular recurrences (39, 42), the WVI strategy, combined with an overdose on the primary disease site, has become the preferred treatment for localized GCTs (42). However, CSI is still necessary for disseminated disease (43).

In an effort to reduce RT doses, Shibamoto et al. (44–46) explored lower doses of 20-24 Gy and achieved comparable outcomes in germinoma patients with negative CSF as well as those with positive CSF or disseminated disease. Neoadjuvant chemotherapy is also justified to shrink tumor size, enabling lower doses and volumes of irradiated fields (47–49). Consequently, focal RT doses of 40-45 Gy are now considered sufficient for treating CNS GCTs with a diameter of 4 cm or smaller (50).

The Children’s Oncology Group (COG) conducted a phase II trial, reducing the dose to 18 Gy WVI with a 12 Gy boost to the tumor bed, for pediatric patients with localized germinoma who achieved complete response after 4 cycles of carboplatin and etoposide (51). Following chemotherapy, complete response (CR) was observed in 62% of patients, partial response (PR) in 30%, and stable disease (SD) in 8%. In a subset of patients with PR or SD who underwent second-look surgery, the CR rate increased to 68%. The 3-year progression-free survival (PFS) and OS rates in this study were 94.5 ± 2.7% and 100%, respectively (51, 52).

Furthermore, the French Society of Pediatric Oncology conducted a study combining chemotherapy (alternating cycles of etoposide/carboplatin and etoposide/ifosfamide) with focal irradiation at 40 Gy (42). They treated 60 patients and reported an 8-year OS of 98% and event-free survival (EFS) of 83%.

Despite the favorable therapeutic outcomes achieved with RT and advancements in technology, it is crucial not to overlook the significant long-term side effects, such as visual deficits, hormonal dysfunction, and neurocognitive decline (53, 54). To address these concerns, PBT has emerged as a promising strategy due to its ability to concentrate radiation dose on the tumor while sparing surrounding healthy tissues (55). This dosimetric advantage not only reduces side effects in the medium to long term but also decreases the risk of radiation-induced secondary malignancies (56). However, the clinical experience with proton radiation is still limited to date.

In our study, we examined 17 patients with germinoma who were treated with neoadjuvant chemotherapy followed by PBT. The cohort characteristics aligned with the tumor epidemiology, with a predominance of males and a median age at diagnosis of 12.19 years (4). Our analysis demonstrated an overall excellent prognosis, with only one relapse among the 17 patients (6%), which was successfully treated with second-line therapy, resulting in the patient being alive at the last follow-up.

The COG phase II trial reported a total of only 8 relapses among 137 eligible patients (52), the same rate found in our experience. These findings confirm that PBT does not compromise local control compared to photon-based RT for similar treatment volumes.

Metastatic central nervous system GCTs are rare. In our cohort, only two patients (12%) had ventricular dissemination at diagnosis, and both cases achieved complete remission of the disease after chemotherapy and CSI, with both patients currently alive without sequelae. Considering the extensive irradiation area required in disseminated cases that necessitate CSI, the advantages of PBT in terms of toxicity and side effect reduction become even more crucial.

In our experience, PBT was well tolerated in all cases with minimal acute toxicity, as previously reported (5).

In our study, we assessed data related to visual, endocrinological, and neuropsychological aspects for all patients. Endocrine dysfunctions are typically attributed to tumor involvement of the hypothalamic-pituitary axis and any neurosurgical interventions. GCT patients have a relatively low risk of developing radiation-induced endocrinopathies. Most endocrinopathies, including hypothalamic dysfunction, were diagnosed before initiating PBT. Radiation doses below 40 Gy rarely result in deficiencies of ACTH, TSH, gonadotropins, and diabetes insipidus (57). GH deficiency is the most commonly associated endocrinopathy with radiotherapy, and its incidence is directly proportional to the hypothalamic-pituitary RT dose (57, 58). Although PBT reduces the risk of endocrine complications, GH deficiency remains the most prevalent new-onset endocrinopathy even after PBT (4). Diabetes insipidus was the most common hormonal abnormality, affecting all children with endocrine abnormalities, and in 9 out of 12 cases, it was accompanied by one or more other endocrinological defects. No new hormonal deficiencies were observed during treatment. GH deficiency was diagnosed in five patients at the last follow-up, consistent with findings in the literature (4). It is worth noting that our observation period was markedly brief to clarify the advantages of PBT in reducing the delayed effects of radiation therapy.

Visual morbidities are primarily associated with the location of the primary tumor. Suprasellar region involvement frequently leads to compression or invasion of the optic chiasm. Additionally, visual deficits can be attributed to the presence of hydrocephalus at diagnosis. In our study, eight patients presented with visual deficits at diagnosis, including three with hydrocephalus, necessitating neurosurgical intervention in two cases. The remaining four patients had tumors located in the suprasellar region. No new-onset visual deficits were detected during treatment, and at the last follow-up, visual deficits persisted in only five patients. Long-term visual decline due to radiation therapy-associated toxicity is rare and typically linked to high radiation doses (> 50 Gy) on the optic pathways (59). PBT does not appear to pose an increased risk of visual impairment (4), as demonstrated in our data.

Neurocognitive impairment is the most prevalent long-term consequence of radiotherapy, with young age, radiation volume, and high doses being identified as major risk factors. The characteristics of PBT appear to decrease the risk of neurocognitive impairment, but currently, there is limited data and follow-up available (5, 60). In a population of sixty patients receiving PBT for different brain tumors (medulloblastoma, gliomas, craniopharyngioma, ependymoma and other CNS tumors) at a mean follow-up of 2.5 years after PBT, no significant change was seen overall in IQ. Moreover, cognitive outcomes did not differ significantly between patients treated with CSI or focal PBT (61).

Our findings indicate no significant changes in cognitive IQ among the subjects examined before and after PBT. Very few data are available to date on neuro-cognitive outcome in patients with brain GCT to compare our results. Although neurocognitive testing was not done consistently, Greenfield et al. reported 14 out of 20 patients to be doing well in school or to have active careers at last follow-up (4).

We emphasize the importance of conducting pre-treatment assessments, as they provide a baseline understanding of cognitive function that remains unaffected by therapies. Monitoring cognitive levels allows for timely and comprehensive rehabilitative interventions if necessary and further clarify the absolute benefit of PBT.

In our series, no secondary malignancies were detected. However, a longer follow-up period is required to fully evaluate this aspect.

The main limitations of our study include the relatively small sample size and the short duration of follow-up. Nevertheless, this study contributes to the limited body of research examining the effects of PBT on disease control, side effects, and neurocognitive evaluations.

## 5 Conclusion

Our findings showcase the potential of PBT as a game-changing treatment for germinomas, offering excellent clinical outcomes while minimizing late toxicities. The promising results pave the way for further exploration of whether PBT can achieve long-term cure rates comparable to standard photon radiotherapy. The future holds tremendous possibilities for this innovative therapeutic approach.

Moreover, the true impact of chemo-radiotherapy’s long-term side effects and the incidence of secondary tumors can only be fully comprehended through extensive follow-up of these patients. This imperative evaluation will solidify the support for PBT as a therapeutic breakthrough in the field.

## Data availability statement

The raw data supporting the conclusions of this article will be made available by the authors, without undue reservation.

## Ethics statement

The study was approved by Institutional Review Board (IRB) of Bambino Gesù Children’s Hospital (RAP- 2023-0008). The study was conducted in accordance with the local legislation and institutional requirements. Written informed consent was obtained from the individuals for the publication of any potentially identifiable images or data included in this article.

## Author contributions

GDB: Data curation, Investigation, Methodology, Writing – original draft. SV: Data curation, Investigation, Writing – original draft. MT: Data curation, Writing – original draft. RA: Data curation, Formal Analysis, Software, Writing – original draft. SL: Data curation, Writing – original draft. EP: Data curation, Writing – original draft. ACac: Data curation, Writing – original draft. GM: Data curation, Writing – original draft. VDR: Data curation, Writing – original draft. ADS: Data curation, Writing – original draft. GA: Data curation, Writing – original draft. SR: Writing – review & editing. GSC: Writing – review & editing. ACar: Writing – original draft, Writing – review & editing. AM: Supervision, Validation, Writing – review & editing. MADI: Writing – review & editing.
